# Supplementary material for: The effects of negative social media connotations on subjective wellbeing of an ageing population: A stressor-strain-outcome perspective
Source: PLoS One. 2024 Jan 30;19(1):e0296973. doi: 10.1371/journal.pone.0296973 (PMC10826960; doi:10.1371/journal.pone.0296973)
Supplement: S1 Appendix — (DOCX) [file pone.0296973.s001.docx]

S1 Appendix. Questionnaire

| **Construct** | **Item Codes** | **Measurement Items** | **Sources** |
| --- | --- | --- | --- |
| Complexity | CMP1 | The functions of social media are not easy to use. | [1] |
|  | CMP2 | It is not easy to get the results that I desire when using social media. |  |
|  | CMP3 | Learning to use social media is not easy for me. |  |
|  | CMP4 | The constant developments and upgrades in the social media are a burden for me. |  |
|  | CMP5 | I feel people younger than me can easily adapt to social media development than me. |  |
| Communication Overload | CO1 | I receive too many messages from friends through social media. | [2] |
|  | CO2 | I feel that I generally get too many notifications from social media while performing other tasks. |  |
|  | CO3 | I waste a lot of my time responding to social media messages with my family and friends. |  |
|  | CO4 | I often feel overloaded with communication from social media. |  |
| Boredom | BM1 | I find it hard to entertain myself. | [3] |
|  | BM2 | I often find myself at “loose ends,” not knowing what to do. |  |
|  | BM3 | Many things I have to do are repetitive and monotonous. |  |
|  | BM4 | In most situations, it is hard for me to find something to do or see to keep me interested. |  |
|  | BM5 | Much of the time, I just sit around doing nothing. |  |
| Privacy Invasion | PIV1 | I feel uncomfortable that my use of this social media can be easily monitored. | [4] |
|  | PIV2 | It bothers me that the information created by my current social media use could be traced even years from now. |  |
|  | PIV3 | I feel that I have to share too much personal information to social media. |  |
|  | PIV4 | I feel that my use of social media makes it easier to invade my privacy. |  |
| Passive Social Media Use | PSMU1 | I am very quiet on social media. | [5,6] |
|  | PSMU2 | I rarely comment on friends’ posts or status. |  |
|  | PSMU3 | I often browse other stories in social media but do not post my own story. |  |
|  | PSMU4 | I rarely interact with others on social media. |  |
| Negative Feelings | While limiting my social media usage | | [7] |
|  | NF1 | I feel negative |  |
|  | NF2 | I feel bad |  |
|  | NF3 | I feel unpleasant |  |
|  | NF4 | I feel sad |  |
|  | NF5 | I feel afraid |  |
|  | NF6 | I feel angry |  |
| Positive Feelings | While limiting my social media usage | | [7] |
|  | PF1 | I feel positive |  |
|  | PF2 | I feel good |  |
|  | PF3 | I feel pleasant |  |
|  | PF4 | I feel happy |  |
|  | PF5 | I feel joyful |  |
|  | PF6 | I feel contented |  |
| Life Satisfaction | LS1 | In most ways my life is close to my ideal. | [8] |
|  | LS2 | The conditions of my life are excellent. |  |
|  | LS3 | I am satisfied with my life. |  |
|  | LS4 | So far I have gotten the important things I want in life. |  |
|  | LS5 | If I could live my life over, I would change almost nothing |  |

References

1. Lee AR, Son SM, Kim KK. Information and communication technology overload and social networking service fatigue: A stress perspective. Comput Human Behav. 2016;55:51–61.

2. Cho J, Ramgolam DI, Schaefer KM, Sandlin AN. The Rate and Delay in Overload: An Investigation of Communication Overload and Channel Synchronicity on Identification and Job Satisfaction. J Appl Commun Res. 2011 Feb;39(1):38–54.

3. Struk AA, Carriere JSA, Cheyne JA, Danckert J. A Short Boredom Proneness Scale: Development and Psychometric Properties. Assessment. 2017;24(3):346–59.

4. Nimrod G. Technostress: measuring a new threat to well-being in later life. Aging Ment Heal. 2018;22(8):1080–7.

5. Chen W, Fan C-Y, Liu Q-X, Zhou Z-K, Xie X-C. Passive social network site use and subjective well-being: A moderated mediation model. Comput Human Behav. 2016 Nov;64:507–14.

6. Zhu X, Bao Z. Why people use social networking sites passively: An empirical study integrating impression management concern, privacy concern, and SNS fatigue. Aslib J Inf Manag. 2018 Mar;70(2):158–75.

7. Diener E, Wirtz D, Tov W, Kim-Prieto C, Choi D. New well-being measures: Short scales to assess flourishing and positive and negative feelings. Soc Indic Res. 2010;97(2):143–56.

8. Lorenzo-Seva U, Calderon C, Ferrando PJ, del Mar Muñoz M, Beato C, Ghanem I, et al. Psychometric properties and factorial analysis of invariance of the Satisfaction with Life Scale (SWLS) in cancer patients. Qual Life Res [Internet]. 2019;28(5):1255–64. Available from: https://doi.org/10.1007/s11136-019-02106-y
