# Supplementary material for: The effects of negative social media connotations on subjective wellbeing of an ageing population: A stressor-strain-outcome perspective
Source: PLoS One. 2024 Jan 30;19(1):e0296973. doi: 10.1371/journal.pone.0296973 (PMC10826960; doi:10.1371/journal.pone.0296973)
Supplement: Attachment — Submitted filename: Response to Reviewers.docx [file pone.0296973.s002.docx]

**Subject: Rebuttal for Manuscript [PONE-D-23-13239]**

Dear Editor,

I trust this document finds you in good health. I would like to extend my deepest appreciation for the valuable time and diligent effort that you and the esteemed reviewers have devoted to thoroughly evaluating my manuscript entitled **"The effects of negative social media connotations on subjective wellbeing of an ageing population: A stressor-strain-outcome perspective."** I appreciate the valuable feedback that has been provided, as it has undoubtedly played a significant role in improving the overall quality of our manuscript.

We thoroughly reviewed and taken into account the comments and suggestions provided by the editor and the reviewers. Significant revisions have been made to the manuscript in order to effectively address the raised concerns. Please find below a comprehensive and detailed response addressing each of the concerns raised.

**Note:** The line numbers provided correspond to the file (containing unmarked version of the revised manuscript.

|  | Journal Requirements | Action/Remarks |
| --- | --- | --- |
| 1 | Please ensure that your manuscript meets PLOS ONE's style requirements, including those for file naming. | The following modifications have been made to fulfil PLOS ONE’s style requirement.  **Title Page**   - The title has been written in sentence case and capitalized only the first word of the title (L 1,2). - Placed the initials of the corresponding author in parentheses after the email address (L11). |
| 2 | Please include captions for your Supporting Information files at the end of your manuscript, and update any in-text citations to match accordingly. | - At the end of the document, provided a caption for the supporting file (L754). - Intext citation is updated accordingly (L284). - File name for the supporting information file is also updated according to the journal’s guidelines. |
|  | Reviewer 1 |  |
|  | The discussion section needs more articulated explanation and rationale for the study. A detail comparative description of different models may be helpful. The method section need further improvement. | In order to improve the rationale of study in within the discussion section, additional discussion has been incorporated (L393-402).  To improve the articulated information and make a clear expression additional literature has been integrated (L68, 84,396, L477-480). We further enlisted the services of native English-speaking professional proofreader (please refer to the revised manuscript with track changes).  A comprehensive comparative description referring to the different implications of SSO model has been provided (see L475-485)  The Method section has been improved with information related to our survey methodology and elucidation of the target population in Malaysia (Refer to the L277-281). |
|  | Reviewer 2 |  |
| 1 | There are no clear difference between the paragraph, causing reading to be exhaustive. | Improved paragraph structure (L58,L98, L135,L150,L160,L352,L459,L467) |
| 2 | L173-174 "People use social media to communicate with friends, so they frequently check their profiles to make sure they don't miss" - do not use short form in academic writing | This particular modification has been executed (L180). However, in order to maintain consistency throughout the document, the manuscript underwent thorough proofreading (please refer to the revised manuscript with track changes). |
| 3 | The development of H6 is not enough. Need to add more. | Additional supportive literature has been added to further cultivate the development of H6 (L238-247) |
| 4 | L290 "Fortunately, we received 399 responses"- no need to mentioned, already stated above, its repetitive. | The number of respondents, 399, was removed from the previous paragraph and subsequently placed at the end (Line 305), thereby ensuring the coherence and sequential integrity of the data collection procedure. |
| 5 | Common method variance need to be tested using full collinearity test, Harman Single Factor test is no longer valid. | To improve the statistical robustness of the data, a full collinearity test (Lines 317-321) was performed, combining a more rigorous statistical approach known as the Variance Inflation Factor (VIF) with the Herman Single factor method. |
| 6 | There is no further discussion on the R2, explained variance except for reporting. | Added extensive discussion concerning R^2^ (Lines 364-374). |
| 7 | Author only refer to . Fu S, Li H, Liu Y, Pirkkalainen H, Salo M. Social media overload, exhaustion, and use discontinuance: Examining the effects of information overload, system feature overload, and social overload. Inf Process Manag. 2020 Nov;57(6):102307 -- what about other studies? | We truly appreciate this valuable input, which has substantially improved the article. Our research was primarily guided by Fu and colleagues' recommendations, but in light of the reviewer's direction, we recognised our study's connection with the recommendations made by Cao et al. (12). Furthermore, as seen in L83-84 and L396-402, the inclusion of other studies provided a vital contextual foundation for our research. Likewise, the incorporation of investigations by Kasim et al. (65) and Tandon et al. (66) has resulted in a significant comparative description,  thereby shedding light on the diverse implications of the SSO model  (Lines 475-485). |
| 8 | L440-441 "There were some limitations in this research if overcome in future research might change the results"- improve and rephrase this sentence. | Improved (Lines 496-497) |
| 9 | L450452 "Experimental method can be helpful to investigate how specific media platforms such as Facebook, Twitter, blogs, and WhatsApp alter attitudes and behaviour in connection to the variables in this study."- Did not mention Tik Tok? | Added TikTok and YouTube (Line 508) |
| 10 | Suggest to refer to these recent related studies in Malaysia  Kasim, N. M., Fauzi, M. A., Wider, W., & Yusuf, M. F. (2022). Understanding Social Media Usage at Work from the Perspective of Social Capital Theory.  Administrative Sciences, 12(4), 170.  Kasim, N. M., Fauzi, M. A., Yusuf, M. F., & Wider, W. (2022). The Effect of WhatsApp Usage on Employee Innovative Performance at the Workplace: Perspective  from the Stressor–Strain–Outcome Model. Behavioral Sciences, 12(11), 456. | Suggested studies have been added referring to reference number 8 and 65 in intext citation and bibliography. |
| 11 | Table 4 is wrong, the beta value is not effect size. It is path coefficient. | Incorporated this correction in Table 4 column 2. |
| 12 | This manuscript require English editing. | We incorporated this recommendation (please refer to the revised manuscript with track changes). |
