# Supplementary material for: The effects of negative social media connotations on subjective wellbeing of an ageing population: A stressor-strain-outcome perspective
Source: PLoS One. 2024 Jan 30;19(1):e0296973. doi: 10.1371/journal.pone.0296973 (PMC10826960; doi:10.1371/journal.pone.0296973)
Supplement: Attachment — Submitted filename: Response to Reviewer.docx [file pone.0296973.s003.docx]

**Subject: Rebuttal for Manuscript [PONE-D-23-13239R1]**

Dear Editor,

I trust this document finds you in good health. I would like to extend my deepest appreciation for the valuable time and diligent effort that you and the esteemed reviewers have devoted to thoroughly evaluating my manuscript entitled **"The effects of negative social media connotations on subjective wellbeing of an ageing population: A stressor-strain-outcome perspective."** I appreciate the valuable feedback that has been provided, as it has undoubtedly played a significant role in improving the overall quality of our manuscript.

I wish to express my gratitude to Reviewer 2 for endorsing the manuscript. Furthermore, I appreciate Reviewer 1 for providing invaluable recommendation regarding the inclusion of support pertaining to the preference of SSO framework. Revision has been made to the manuscript in order to incorporate the valuable suggestion.

**Note:** The line numbers provided correspond to the file (containing unmarked version of the revised manuscript.

|  | Reviewer 1 |  |
| --- | --- | --- |
|  | Please share how you explored other theories and decided to focus on the stressor-strain-outcome (SSO). | In order to improve the significance of SSO framework in our study, supportive discussion has been incorporated (L135-144). |
